# Pathological changes of the anatomical structure and markers of the limbal stem cell niche due to inflammation

**Published:** 2013-02-26

**Authors:** Mario Nubile, Claudia Curcio, Harminder S. Dua, Roberta Calienno, Manuela Lanzini, Manuela Iezzi, Rodolfo Mastropasqua, Luca Agnifili, Leonardo Mastropasqua

**Affiliations:** 1 Ophthalmology Clinic University “G. d'Annunzio” of Chieti and Pescara, Italy; 2 Division of Ophthalmology and Visual Sciences, University of Nottingam, UK; 3 Department of Medicine and Ageing Science, UO Experimental Pathology, CeSI, University “G. d'Annunzio” of Chieti and Pescara, Italy; 4 Ophthalmology Clinic University of Verona, Italy

## Abstract

**Purpose:**

The corneoscleral limbus is the site of corneal epithelial stem cells (SC). The aim of this study is to evaluate the expression of different SC markers in the normal human limbus and to determine how this is affected by inflammation.

**Methods:**

Corneoscleral specimens from healthy and inflamed donor eyes were examined by immunohistochemistry/immunofluorescence for p63, vimentin, laminin 5, integrin α6, β1, β4, ABCG2, desmoglein 3, connexin 43, N-cadherin, and cytokeratins 12 and 15. The distribution and anatomic structure of the limbal crypts and the percentage of SC marker antigens in healthy donors were analyzed. In inflamed tissues, we evaluated the anatomic structure of the limbal epithelial crypt (LEC) and the positivity for SC markers.

**Results:**

In normal limbus, the niche structures were distributed differently. The variability of their number correlated with the percentage of p63 positivity. Integrin β1 staining directly correlated with p63 positivity while the remaining proteins were variably and widely distributed. Double staining for p63 and vimentin did not reveal any co-localization. In inflamed eyes, the basal cells in the crypts were “stretched” and surrounded by inflammatory cells, and only a few SC markers were still present.

**Conclusions:**

Diseases involving the limbus may result in marked changes of expression of SC markers within the LEC and also alter the crypt structure.

## Introduction

A specialized local microenvironment or niche, which regulates the maintenance, self-renewal, activation, and proliferation of stem cells (SC) via external signals, is one of the key prerequisites for stem cells’ function and fate [1]. The ocular surface corneal epithelial stem cells are located at the sclerocorneal limbus in the palisades of Vogt and are highly pigmented structures with an abundance of melanocytes, antigen presenting cells, and lymphocytes [2,3]. Recently Dua and colleagues identified an anatomically defined site in the human limbus, which they termed the limbal epithelial crypt (LEC), that could serve as a niche [2]. Limbal epithelial stem cells (LESC) are anatomically situated in specific niches. They are well-defined structures that are located deep in the substantia propria of the limbus, thus providing protection and regulation as well as a microenvironment comprising an extracellular matrix with a multitude of resident cells [2,3]. Stem cell niches have been identified in several human locations, such as intestinal crypts, hair follicles, and bone marrow [4,5]. The limbal “niche” represents the site where the LECs are protected from the external environment. It is surrounded by other non-stem cells, which provide the signals required to maintain their stemness [5]. Their location and distribution are not regular along the limbal circumference. Niches are predominantly present in the nasal region and the mid or distal limbus at the conjunctival end [6].

Several molecules have been proposed as epithelial limbal SC markers, but no single marker unequivocally identifies them. A distinction is made between negative and positive markers. Negative markers are those that are absent or poorly expressed at the limbus, like the keratin pair K3/K12 found in the central cornea, connexin 50 [5], and involucrin [3]. Positive markers are integrins (cell adhesion molecules), in particular β1, β4, and α6, p63 (transcription factor), vimentin (intermediate filament protein), ABCG2 [ATP-binding cassette (ABC) transporters], connexin 43 (gap junction protein), laminin 5 (non-collagenous basement membranes), and desmoglein 3 (cell membrane protein) [2-5,7-12].

Multiple etiologies can induce severe damage to the limbal epithelial stem cells, such as aniridia, chemical burns, multiple ocular surgeries, severe microbial infection, Steven-Johnson syndrome, and ocular cicatricial pemphigoid. When LSC deficiency occurs, ingrowth of the conjunctival epithelium with goblet cells on the cornea (conjunctivalization of the cornea), chronic inflammation, and neovascularization are the key pathological signs [13]. Loss of the palisades of Vogt, late fluorescein staining of areas of the conjunctivalized cornea, and persistent epithelial defects are regarded as important clinical features. It is thought that ocular surface inflammation affecting the limbal region exerts significant effects on limbal stem cell function as well as the limbal niche and its micro-environment [14]. Inflammatory cell infiltration was found in corneal pannus specimens removed from patients with total LSC deficiency, thus supporting the notion that inflammation plays a major role in the development or maintenance of LSC deficiency [15]. The aim of the present investigation is to evaluate the expression of different SC markers in the normal human limbus and to determine how inflammation affects the expression of these antigens.

## Methods

### Samples preparation

The study adhered to the tenets of the Declaration of Helsinki. The protocol used was approved by our institutional review board. Eight human eye bank corneal buttons with scleral rims not suitable for transplantation (ages ranged from 52 to 80 years; mean age 72.3±10.3 years) were included in the study. The average death to enucleation time was 8 h (range from 4 h to 10 h). The mean storage (Eusol-C, AlchimiaSrl, Pordenone, Italy) time between eye bank procedures and fixation was 26 h (range from 20 h to 48 h).

In addition, five pathological corneo-scleral tissue samples (ages ranged from 59 to 85 years; mean age 69.4±9.6 years) were collected at the time of enucleation of the eye due to uncontrolled infectious endophthalmitis affecting the cornea and the ocular surface and immediately fixed after retrieval. The etiology of endophthalmitis was originally related to corneal infection and included *Stenotrophomonas maltophilia, Aspergillus flavus, Candida albicans, Pseudomonas aeruginosa*, and one case of HSV necrotizing keratitis with cornea perforation and subsequent unidentified microbial superinfection. All pathological eyes presented variable degrees of limbal inflammation.

For normal donors, no evidence of any disease, desiccation, or damage was noted. All tissues were fixed in 4% formalin (Bio Optica, Milano, Italy).

**Figure 1:**
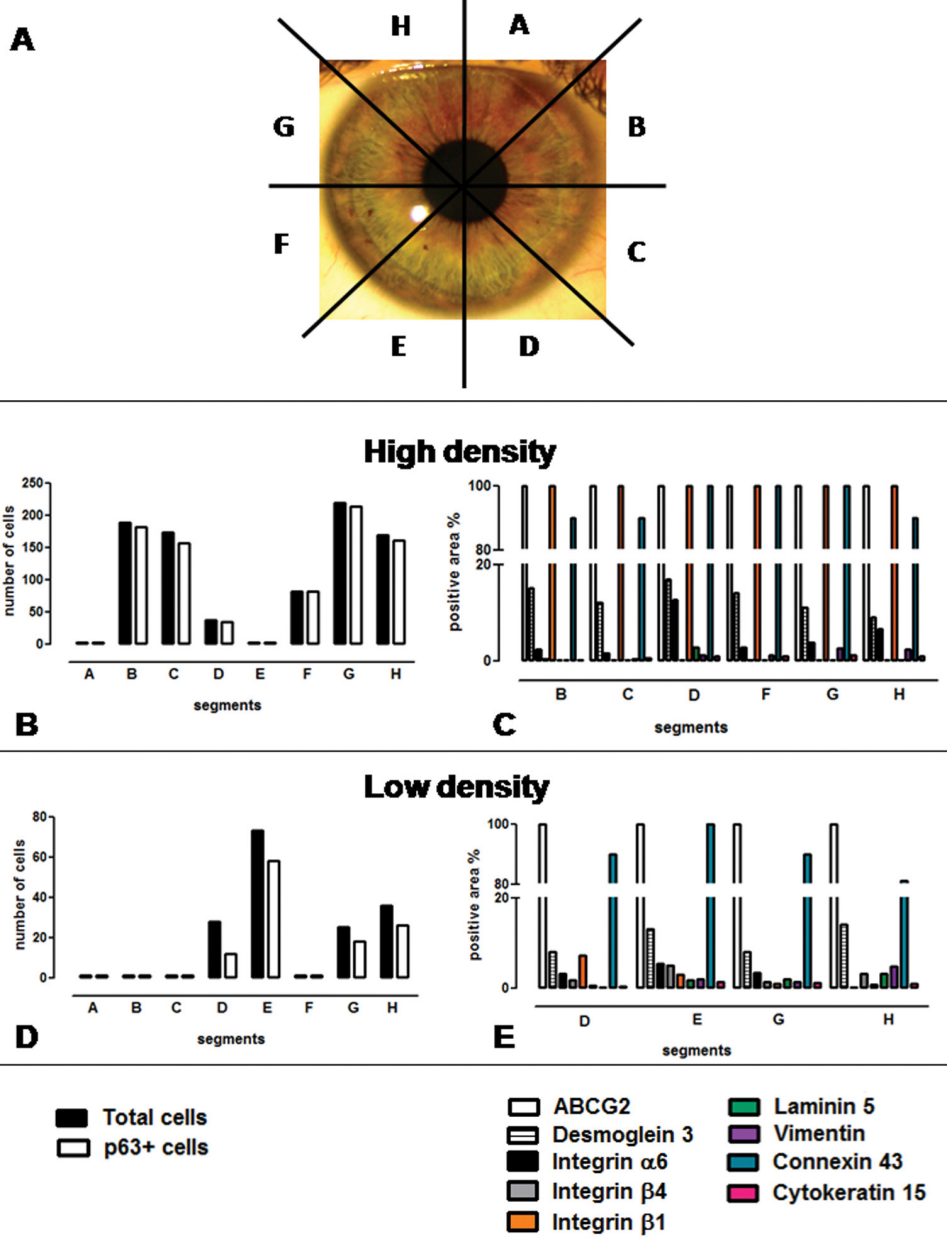
A To evaluate the presenceof crypts, the entire corneoscleral ring was divided into eight segments (from **A** to **H**), and an hematoxylin and eosin staining was done. **B**-**D**: The presence and number of cells in the crypts for each segment were evaluated (black bars). In the segments in which we found cells, p63 staining was done (white bars). According to the presence and number of crypts observed within the entire corneoscleral rings in each of the segments and the positivity of p63 cells in the LECs, the samples were divided into two groups, defined as High (**B** and **C**) and Low Density (**D** and **E**). The differences in the number of p63 positive cells between the High and Low Density groups were significant (p<0.0001; Student t test; High group n=43 versus Low groupn=14). **C**-**E**: The distribution of limbal stem cell markers in Low and High Density groups. In segments with p63 positive cells, other limbal stem cells markers were evaluated, and the results were expressed as a percentage of positive areas in the crypt. Representative graphs with the distribution of markers in the High (**C**) and Low (**E**) groups are shown. ABCG2 (white bars) and connexin 43 (blue bars) remain highly expressed in both groups. Integrin β1 (orange bars) decrease in the Low Density groups. Desmoglein 3 (striped bars), integrin α6 (black bars), integrin β4 (gray bars), laminin 5 (green bars), vimentin (violet bars), and cytokeratin 15 (pink bars) were variably 400 expressed.

Healthy donors were divided into eight equal segments (Figure 1A), and all samples were then embedded in paraffin (Bio Optica). According to a previously published protocol [6] with minor modifications, 3 µm sections of the blocks were serially cut with a microtome (Leica Microsystems GmbH, Wetzlar, Germany) and monitored for the presence of LEC by staining every 10^th^ to 15^th^ section with hematoxylin and eosin stains. When a region containing the LEC was identified, the adjacent preceding and subsequent sections were collected for immunohistochemistry and immunofluorescence staining. Progressing from the origin of each identified crypt, once the maximal extension was observed, the number of epithelial cells contained in the corresponding section was counted.

### Immunohistochemistry

Immunohistochemistry analysis was performed on sections in which we observed the presence of LECs by hematoxylin and eosin staining and for all inflamed samples. Each section was immune-stained with the following antibodies: p63 (1:200; Dako, Glostrup, Denmark), vimentin (1:25,000; Dako), laminin 5 (1:25; Dako), connexin 43 (1:20; Zymed Laboratories, Inc., San Francisco, CA), desmoglein 3 (1:50; Zymed Laboratories, Inc.), integrin α6 (1:10,000; Santa Cruz Biotechnology, Santa Cruz, CA), integrin β1 (1:50; Santa Cruz Biotechnology), integrin β4 (1:1,000; Santa Cruz Biotechnology), ABCG2 (1:20; Abcam, Cambridge, UK), and cytokeratin 15 (1:2,000; Thermo Scientific, Watham,MA). Formalin fixed tissues were deparaffinized and pretreated by microwave antigen retrieval using buffered EDTA pH9 (required for p63, vimentin, cytokeratin 15, and laminin 5) and buffered sodium citrate pH6 (for ABCG2). For all of these antigens, with the exception of integrin α6 for which the ABC technique was used, the En Vision system (Dako) was used before diaminobenzidine tetrahydrochloride (Dako) incubation. A negative control was performed for each antigen using the specific isotype antibody as control. All slides were stained for the same antigen together with the same antigen retrieval buffer, where required, and the same antibody dilution. For double immunohistochemistry staining, Ferangi Blue Chromogen Kit (Biocare Medical, Concord, CA) was used to identify integrin α6, β1, or β4 while for p63, diaminobenzidine tetrahydrochloride was the chromogenic substrate. It was not possible technically to perform double immunoistochemical staining for p63, desmogelin 3, and laminin 5 in our study due to issues related to isotype and pretreatment.

### Fluorescent staining

After pretreatment with microwave, sections were incubated with their respective antibodies, p63 (diluted 1:50) and vimentin (diluted 1:15,000), overnight (ON) at 4 °C. After washes, IgG2a Alexa Fluor 488 (1:200, Molecular Probes, Eugene, OR) or IgG1 Alexa Fluor 546 (1:200, Molecular Probes) were incubated for p63 and vimentin, respectively. Finally, to stain nuclei, DRAQ5 (1:150; Alexis Corporation LDT, San Diego, CA) was used. To evaluate the positivity for cytokeratin 12 (Santa Cruz Biotechnology) in inflamed tissues, slides hydrated with distilled water and RNase A (Sigma-Aldrich, St Louis, MO) diluted 1:300 in phosphate buffered saline were incubated at room temperature. The specimens were washed and phosphate buffered saline-bovine albumin solution 1% was added for 1 h at room temperature. Finally, cytokeratin 12 antibody diluted 1:50 was incubated at 4 °C. Samples were washed and anti-goat Alexafluor 488 (Molecular Probes, Eugene Oregon, MN) diluted 1:200 and propidium iodide (Invitrogen, San Giuliano Milanese, Italy) at 1:150 were added and incubated for 1 h at room temperature. Samples were mounted with a drop of Fluorescent Mounting Medium (Dako, Glostrup, Denmark), and Zeiss Confocal LSM 510 (Carl Zeiss MicroImaging GmbH, Vertrieb, Germany) was used to visualize the cells.

### Image and statistical analysis

Adobe Photoshop (Adobe Systems Inc., Jose, CA) was used to evaluate the total and positive pixels of each crypt for cytoplasmatic staining (vimentin, laminin 5, integrin β1, integrin β4, integrin α6, ABCG2, desmoglein 3, and connexin 43) while for nuclear staining (p63), positive cells were counted. Differences in markers expression for selected antigens were assessed by using the Student *t* test (GraphPad Prism 5, GraphPad Software, San Diego, CA).

## Results

### Normal limbus

All eight corneo-scleral rims demonstrated limbal epithelial crypts, and a total of 71 LECs were identified (numbers of crypts range 6–12; mean 8.87±2). In normal corneo-scleral rims, LECs were not identified in all of the segments. According to the density of the LECs observed in the donor corneo-scleral rims, the samples were subdivided into two groups termed as “High Density,” which included four out of the eight samples, and “Low Density,” including the remaining four samples, as shown in Table 1. The High Density group included corneo-scleral rims with five or more segments containing LECs with a high number of cells (Figure 1B, black bars) whereas, in the corneo-scleral rims included in the Low Density group, less than five segments containing smaller crypts formed by a small number of cells were observed (Figure 1D). The number of crypts observed in the different donors and the number of cells observed in the crypts did not correlate with age or gender.

**Table 1:** Samples classification: the number of segments containing limbal epithelial crypts, the total number of crypts, and the number of cells within the crypts in all of the samples studied.

Sclerocorneal rims			
High Density	Segments containing crypts	Number of crypts	p63+/total counted cells in crypts (percentage of positive cells)
1	6	9	800/850 (94.1%)
2	7	12	552/570 (96.8%)
3	5	10	555/590 (94.1%)
4	6	11	991/1180 (84%)

Low Density			
1	4	6	122/170 (71.7%)
2	4	8	111/230 (48.2)
3	3	7	135/215 (62.8%)
4	4	8	155/260 (59.6%)

### Difference in p63 distribution

To confirm the stem cell characteristics of the cells observed within the identified crypts, p63 staining was done. The difference in the number of cells expressing p63 was statistically significant between the two groups (p<0.0001, Student *t* test). In the High Density group, almost all cells were p63 positive (Figure 1B, white bars). Conversely in the Low Density group, only partial p63 positivity was observed (Figure 1D, white bars).

### Correlation between positivity of p63 and other markers

In the segments with p63 positive cells, the presence of vimentin, laminin 5, integrin β1, integrin β4, integrin α6, ABCG2, desmoglein 3, cytokeratin 15, and connexin 43 markers was evaluated as a percentage of positive areas in the crypts. Interestingly, the positivity for integrin β1 was strongly evident in the crypts belonging to the High Density group while it decreased significantly in the Low Density group (p=0.0081; Student *t* test) corresponding to p63 (Figure 1C,E). On the other hand, ABCG2 and connexin 43 were highly expressed in the High and Low Density groups, as shown in Figure 1C,E. The other antigens showed a low to moderate variable expression in both groups (Table 2).

**Table 2:** Mean expression of limbal epithelial stem cell markers in high and low density crypts

Sclecorneal rims	Limbal epithelial crypt markers (mean percentage of positive area/total area)								
	Integrin α6	Desmoglein 3	ABCG2	Integrin ß1	Integrin ß4	Laminin 5	Connexin 43	Vimentin	Cytokeratin 15
High Density	3.23	14.4	100	99	1.7	0.97	95	1.8	1,1
Low Density	2.75	13.25	100	5.55	2.1	1.2	90	2	1,5

### Antigen co-expression

**Figure 2:**
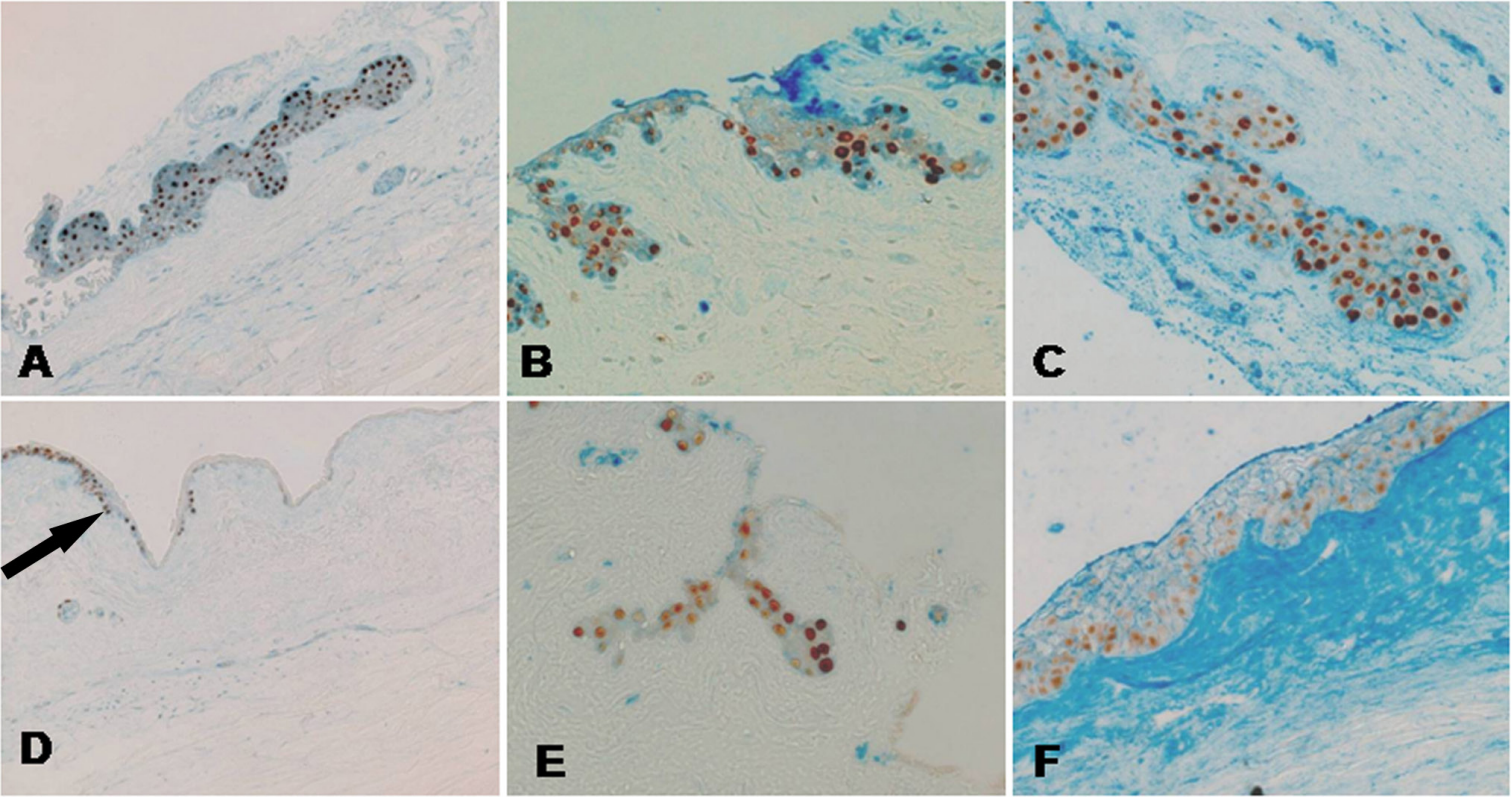
Integrin β1 correlates with p63 positivity. All images are oriented with the epithelial side up and the stromal side down. **A-D**: Double immunohistochemistry staining for integrin β1 (blue) and p63 (brown) showed the decreased expression of integrin β1 and p63 (arrow) in the Low (**D**) as compared to the High group (**A**). The difference in integrin β1 expression was significant (p=0.0081; Student *t* test; High group n=43 versus Low group n=14). The image was magnified 200 times. **B-C-E-F**: p63 correlates with integrin β1 but not with integrin β4 and integrin α6. No statistical difference was observed in the distribution of the integrin β4 (blue) and p63 (brown) panels (**B** and **E**) and the integrin α6 (blue) and p63 (brown) panels (**C** and **F**) in the two groups. The image was magnified 400 times.

**Figure 3:**
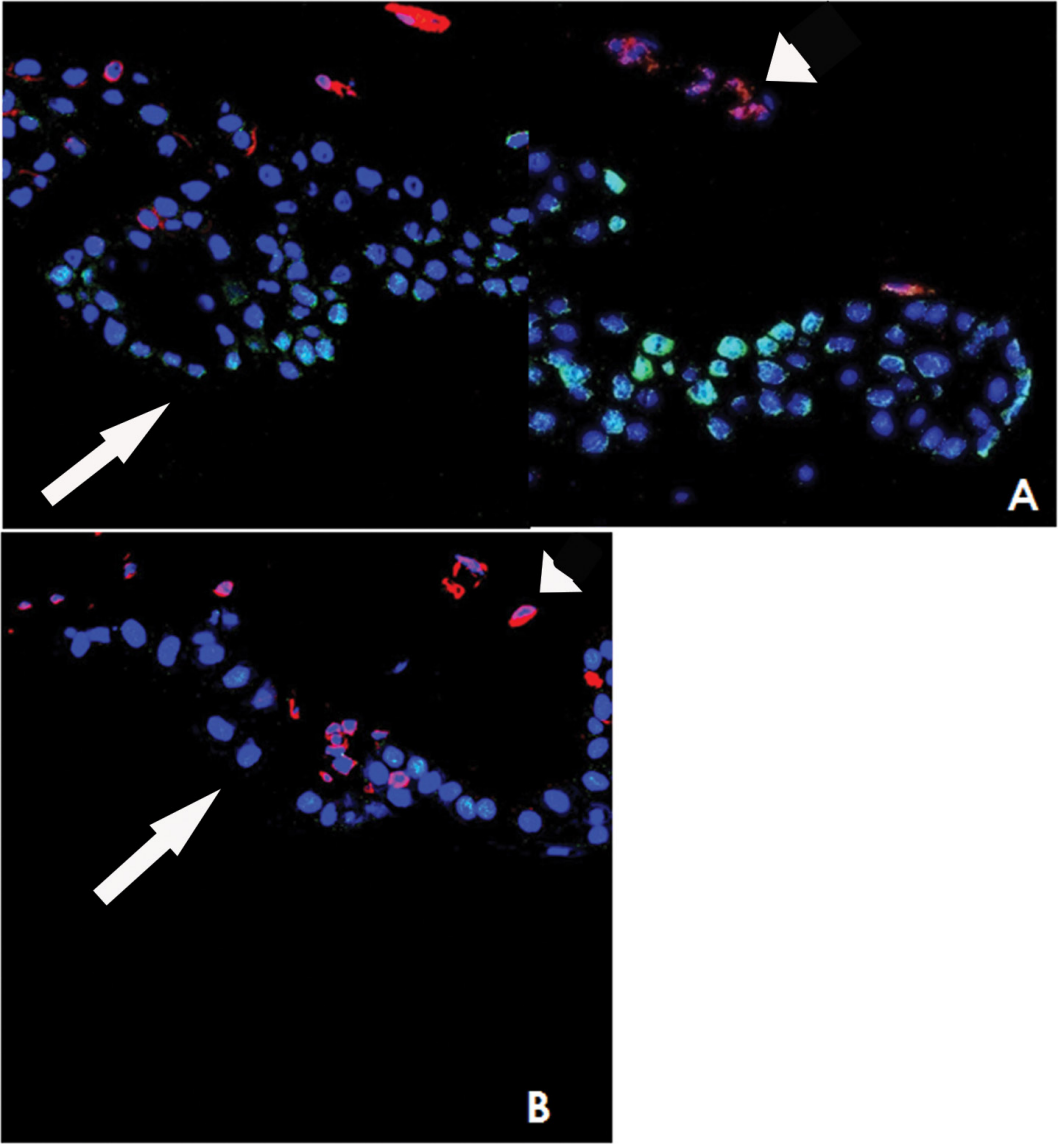
Confocal microscopy images of High (**A**) and Low (**B**) density crypts were stained for p63 (green), vimentin (red), and nuclei (blue). Vimentin positive cells were in the basal layer of crypt (arrow head), and p63 positive cells were in the superficial layer (arrow), which was always negative for vimentin. The image was magnified 630 times.

The antigens that showed a different distribution in the crypts in the two groups, like integrins β1, and those antigens that were similarly expressed in both groups (integrin β4, α6, and vimentin), were stained together with p63 to evaluate their co-expression. As shown in Figure 2A, p63 co-localized with integrin β1 in all cells in the High Density group, Conversely, in the Low Density group, in which there was a significant reduction of integrin β1 positivity, only a few cells co-expressed the two antigens (Figure 2D). p63 partially co-localized with integrin β4 (Figure 2B,E) and integrin α6 (Figure 2C,F) in both groups with no significant difference. Vimentin did not co-localize with p63 and was present mainly in the basal cells of the crypts, as shown in Figure 3.

### Pathological cases

**Figure 4:**
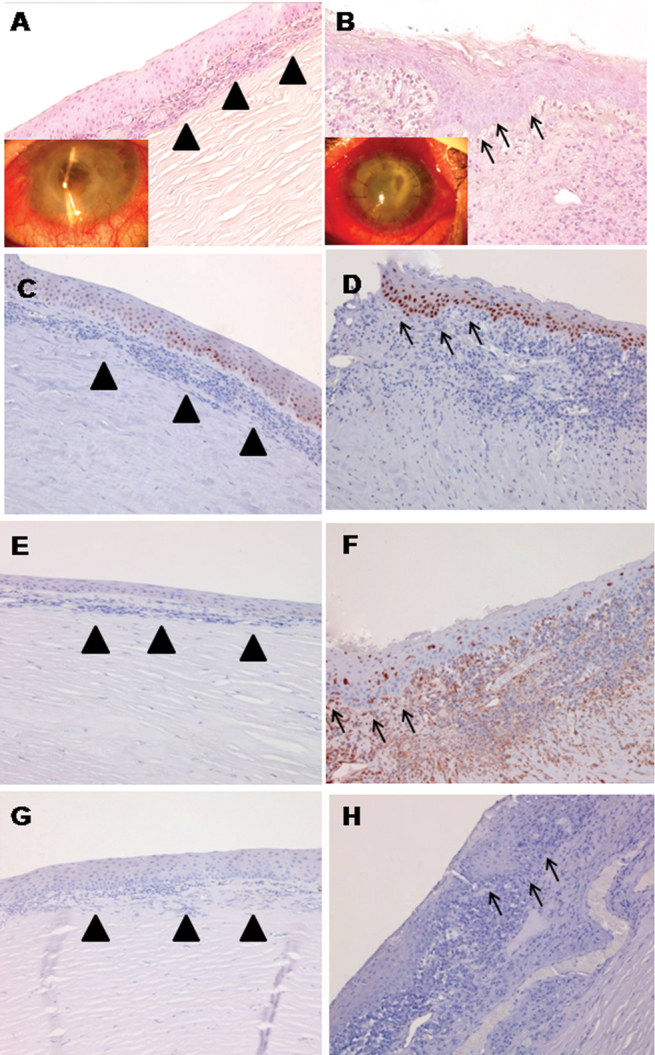
All images are oriented with the epithelial side up and the stromal side down. Examples of tissue with a small (**A**, **C**, **E**, **G**) and highnumber of inflammatory cells (**B**, **D**, **F**, **H**) are shown. Haematoxylin and eosin staining show the morphology of crypts with a small (**A**) and high (**B**) number of inflammatory cells. The arrow heads indicate the subepithelial infiltration of the inflammatory cells while the arrows take the shape of the crypts profile surrounded by inflammatory cells. Inserts: p63 positive cells were evident in the lower and middle layers of the epithelium in both inflamed tissues (**C** and **D**). Few vimentin positive cells were observed in the samples with a high number of inflammatory cells (**F**) while they were absent in the other one (**E**). Integrin β4 staining was negative (**G** and **H**) in both inflammatory conditions. The images were magnified 200 times.

The pathological tissues were analyzed according to the presence of inflammatory cells. In two cases (HSV and *Candida albicans* infection), few inflammatory cells (less than 200 cells/field) were observed while in the other three samples (*Stenotrophomonas*, *Aspergillus*, and *Pseudomonas*) more than 500 inflammatory cells/field were found. Phenotype analysis of the inflammatory cells by immunohistochemistry revealed that, in all the tissue samples analyzed (data not shown), they were predominantly macrophages, granulocytes, and lymphocytes. In one case only, the anatomy of the crypts was completely disrupted. No deep crypts were identified in any of the sections of the sample, and few inflammatory cells were observed in the limbal subepithelial region (Figure 4A). In the other pathological cases, crypts were still present but had an atypical structure: they were smaller and their basal cells were “stretched” in an elongated shape compared to the morphology observed in the normal limbus. They were surrounded by inflammatory cells while a basal membrane was not clearly detectable (Figure 4B, arrows). Tissues were also analyzed for SC antigens, and the expression of the different markers observed in the pathological tissues is reported in Table 3.

**Table 3:** Expression of limbal epithelial crypt markers in normal and pathological tissues

Parameters	Normal limbus	Small number of inflammatory cells (two cases)	High number of inflammatory cells (three cases)
p63	+	+	+
ABCG2	+	+	+
Desmoglein 3	+	+/−	-
Vimentin	+	+/−	+
Integrin β1	+/−	+/−	+/−
Integrin β4	+	-	-
Integrin α6	+	-	+/−
Laminin 5	+	+/−	+/−
Connexin 43	+	+	+
Cytokeratin 15	+	+	+

**+**=moderate to high expression; **+/−**=low to moderate expression; **-**=no expression

**Figure 5:**
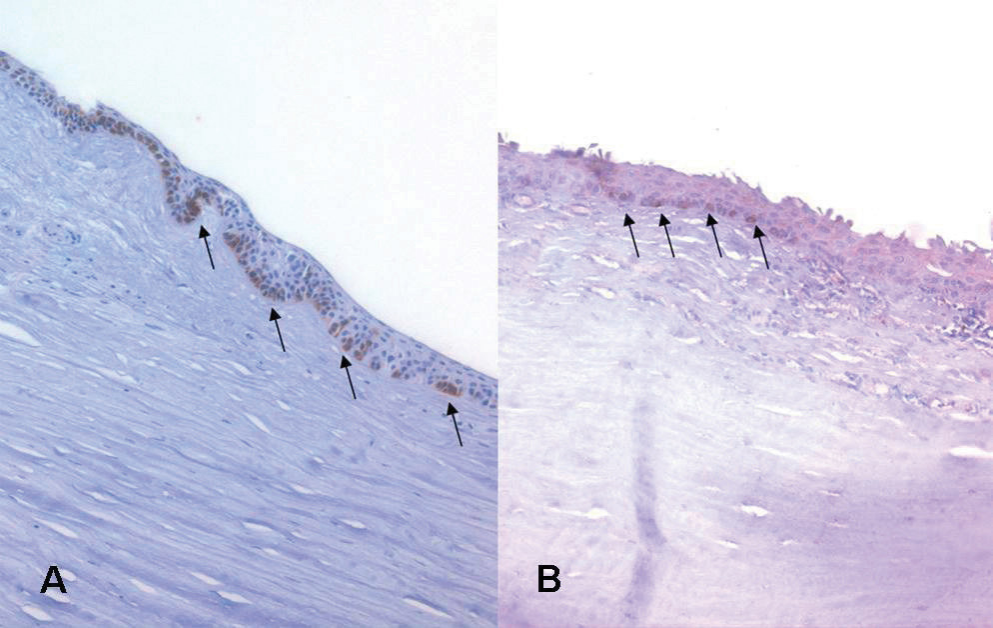
The basal cells of the limbalcrypt are positive for cytokeratin 15 (arrows) both in the healthy (**A**) and inflamed (**B**) tissues. The images were magnified 200 times.

p63 (see Figure 4C,D), ABCG2, cytokeratin 15 (Figure 5B), and connexin 43 maintained an evident expression in all tissues analyzed despite the mild to severe limbal inflammation and the disappearance and/or morphological alterations of the recognizable crypts whereas the expression of integrin β4 was extremely reduced in both severely and less inflamed tissues (Figure 4G,H). The expression of other antigens, such as vimentin (Figure 4E,F), appeared variable as shown in Table 2. Positivity for cytokeratin 12 was always evident in the central cornea, confirming that normal differentiated corneal epithelial cells persisted despite the (partial) limbal stem cell deficiency noted clinically and histologically.

## Discussion

The corneal epithelium is maintained by a pool of SC that resides predominantly in the corneo-scleral limbus. The distribution of SC is not uniform along the limbus but appears to be concentrated in defined anatomic structures termed the limbal epithelial crypt [2,4,6]. Partial or total limbal SC deficiency resulting from damage to the limbus continues to pose a challenge in ocular surface reconstruction procedures aimed at restoring sight. Despite several studies, there is no single marker that can identify a limbal SC, and reliance is placed on a combination of different markers, some negative and some positive [8]. Our results obtained from the LECs of normal eyes suggest that the phenotype of limbal SC should be p63+/ABCG2+/vimentin+/desmoglein 3+/connexin 43+/integrin α6+/integrin β4+/integrin β1+/−, and laminin5+. Of these p63, ABCG2, integrin β1, and connexin 43 were the ones most highly expressed. The absence (negative staining) of connexin 43 has been regarded as a characteristic of SC [16]. However more recent studies [4,6] have demonstrated that the LECs, the palisades with LECs, and the adjacent limbus show connexin 43 positivity compared to other areas of the limbus where this is negative [4,6]. Studies on hematopoietic SC have demonstrated that connexin 43 expression is upregulated among niche cells and is associated with SC growth [17,18].

Like others [4-6], we too found connexin 43 in relation to both low and high density LEC supporting the suggestion that, in the human limbus, connexin 43 is likely to be associated with SC niches. Furthermore, the total number of LECs we counted in our eight normal samples was similar (71 versus 74) to that previously reported [6]. In our study, we refined this observation by undertaking a count of cells expressing SC markers within the LECs and found that corneo-scleral rims with a greater number of LECs also had a higher density of cells within the LECs. We propose this to be a normal variation in individuals. However, it was interesting to observe that the low cell counts within the LECs correlated with the decreased expression of the SC marker p63. Pellegrini et al. [7] have shown that p63, a transcriptional factor involved in morphogenesis, is a keratinocyte stem cell marker of the corneal epithelium and epidermis on the basis of the clonogenic capacity of the cells. Thus our results would suggest that there is a variation in the number and distribution of limbal SC in different individuals with some possessing reduced proliferative potential in their limbus compared to others.

To substantiate the above conclusion, we studied the expression of other known SC markers in the regions where p63 positive cells were observed. Interestingly, we found that the expression of some of these mirrored the expression of p63 while others did not.

Integrins are adhesion molecules that mediate cell-matrix or cell-cell adhesion and transduce signals that regulate gene expression, cell growth, and migration [19,20]. Zhou et al. [21] observed that the functional properties of stem-like corneal epithelial cells were enhanced when co-cultured with embryonic stem cells via activation of the integrin β1-FAK-PI3K/Akt signaling pathway. These cells also showed increased expression of ABCG2 and p63. Similarly, analyzing antigen co-expression in our study, integrin β1 co-localized with p63 in all cells in the High Density LECs while this co-expression was significantly reduced in the Low Density LECs, suggesting that in the latter crypts, we had both decreased proliferative potential (reduced p63 positive cells) and reduced survival (few integrin β1 positive cells).

ABCG2, a member of the ATP binding cassette transporters, has been identified as a molecular determinant for bone marrow stem cells and proposed as a universal marker for stem cells [11,22]. This did not mirror the distribution of p63 as ABCG2 expression was similar in both the High and Low Density crypts.

The basement membrane is considered to be part of the SC niche that can influence the behavior of SC, and basement membrane heterogeneity has been demonstrated in relation to corneal and limbal epithelia [8,23]. Our analysis of the expression of laminin 5 revealed that this marker was present in the basement membrane of both types of crypts. Similarly, other adhesion molecules, such as integrin β4 and integrin α6, were also expressed in both the High and Low Density crypts.

Cytokeratin 15 expression occurs at an early stage of keratinocyte differentiation, and its expression in limbal SC decreases with serial passage. This observation has also been described in actively proliferating keratinocytes [24]. We found that cytokeratin 15 was mainly expressed in the basal layers of both the High and Low Density LECs.

Vimentin is an intermediate filament that is expressed in basal and suprabasal cells [6,8,12] and is absent in the central cells of the LECs [6], which we also found in our study. The absence of cells with the vimentin+/p63+ phenotype in the LEC and the presence of p63+ cells in the central cornea suggests that vimentin is a marker of less differentiated cells [6]. Desmoglein 3, a cell adhesion molecule, is a negative marker of SC [4]. We found desmoglein 3 in the superficial cells and other negative markers in the basal layers of the LEC, suggesting that the SC are likely to be located in the basal cells of the LEC.

Thus from the perspective of the normal limbus and LEC, we have demonstrated the presence of High and Low Density LECs with differential expression of the known SC markers. Although it is tempting to postulate that this represents a differential proliferative potential of the cells in the LEC, further studies are required to confirm this.

The fate of the LEC in relation to limbal stem cell deficiency has not previously been explored. Moreover, the effect of inflammation on limbal SC in general and on the LEC in particular is also not known. We had the opportunity to explore these questions in five human eyes that had to be removed as a result of severe infection-related inflammation. We were able to explore the entire corneo-scleral circumference, as was done with the normal samples described above. The scoring of positively staining tissue was done using stain intensity rather than a percentage of the positive area, as distinct crypts were not found in all samples.

In the pathological limbal tissues, the density and morphology of the LECs was altered. In the majority of cases, the crypts were still visible, although they presented an atypical morphology that was smaller, characterized by elongated-shaped cells, had a less-evident basement membrane, and was surrounded by inflammatory cells infiltration of lymphocytes, granulocytes, and macrophages. These observations suggest that limbal inflammation is associated with detectable changes in the structure of the LECs.

It is already known that ocular surface inflammation plays a negative role in the survival of transplanted limbal epithelial stem cells and that chronic inflammation exerts a negative influence on the maintenance and function of the resident limbal stem cells [25]. Although none of the pathological eyes studied presented clinical signs of limbal stem cell deficiency, morphological changes of the LEC structure were observed. A corresponding alteration in the pattern of expression of the known SC markers was also seen in the LEC.

Previous studies on normal limbal epithelial cells showed a high expression of ABCG2 and ΔNp63, together with an enhanced proliferative capacity [11,26]. Inflamed tissues were positive for ABCG2, cytokeratin 15, and p63, despite the grade of inflammation suggesting that LECs have an inherent capacity to resist inflammation-induced damage as a survival mechanism.

Interestingly, connexin 43 positivity was constantly observed in the pathological tissues. In addition to the role in SC biology discussed above, connexins are also involved in a range of non-gap-junctions functions (suppression of cells, tumor growth, differentiation, and migration) [6]. Connexin 43 positive cells in inflamed tissues may have a role in suppressing inflammation-induced cell damage. Compared to the findings seen in normal tissue, laminin 5 was reduced in inflamed tissue suggesting that this corneal basement membrane protein can at least be partially responsible for the maintenance of the stemness of SC. Desmoglein 3 was negative only in the presence of a severe inflammatory cell infiltration, possibly indicating the reduced capacity of the surviving cells to form desmosomes and maintain cell-cell adhesion. Vimentin, as a marker of less differentiated cells, was always positive, suggesting that SC have a survival advantage in the presence of inflammation.

In inflamed tissue, we observed a marked reduction in the expression of integrin β4 compared to integrins β1 and α6. A negative regulatory role has been attributed to integrin β4 in the response of human lung endothelial cells to inflammation [24]. Loss of β4 in inflamed LEC may be associated with damage to SC.

All pathological tissues examined showed the presence of cytokeratin 12 in the central corneal epithelium, suggesting that, at the time of analysis, none of the cases showed features of limbal stem cell deficiency or conjunctivalization of the corneal surface. A previous study has shown that central islands of corneal epithelia can survive for long periods of time in the presence of severe limbal damage [27]. The researchers hypothesized that the central basal epithelium is capable of sustaining the central epithelial cell mass. Our study shows that even when the limbal architecture is damaged by inflammation, the limbal SC survive, especially in the LEC.

To the best of our knowledge, this study provides the first illustration of the effect of inflammation on the corneo-scleral limbus and the LEC. Such studies will help us understand the mechanisms involved in the survival and destruction of SC in response to inflammation.

## References

[1] Schlötzer-Schrehardt U, Dietrich T, Saito K, Sorokin L, Sasaki T, Paulsson M, Kruse FE (2007). Characterization of extracellular matrix components in the limbal epithelial stem cell compartment.. Exp Eye Res.

[2] Dua HS, Shanmuganathan VA, Powell-Richards O, Tighe PJ (2005). Joseph. A Limbal epithelial crypts: a novel anatomical structure and a putative limbal stem cell niche.. Br J Ophthalmol.

[3] Li W, Hayashida Y, Chen YT, Tseng SCG (2007). Niche regulation of corneal epithelial stem cells at the limbus.. Cell Res.

[4] Yeung AM-H, Schlotzer-Schrehardt U, Kulkarni B, Tini NL, Hopkinson A, Dua HS (2008). Limbal epithelial crypt A model for corneal epithelial maintenance and novel limbal regional variations.. Arch Ophthalmol.

[5] Pajoohesh-Ganji A, Stepp MA (2005). In search of markers for the stem cells of the corneal epithelium.. Biol Cell.

[6] Shanmuganathan VA, Foster T, Kulkarni BB, Hopkinson A, Gray T, Powe DG, Lowe J, Dua HS (2007). Morphological characteristics of the limbal epithelial crypt.. Br J Ophthalmol.

[7] Pellegrini G, Dellambra E, Golisano O, Martinelli E, Fantozzi I, Bondanza S, Ponzin D, McKeon F, De Luca M (2001). p63 identifies keratinocyte stem cells.. Proc Natl Acad Sci USA.

[8] Schlötzer-Schrehardt U, Kruse FE (2005). Identification and characterization of limbal stem cells.. Exp Eye Res.

[9] Shortt AJ, Secker GA, Munro PM, Khaw PT, Tuft SJ, Daniels JT (2007). Characterization of the limbal epithelial stem cell niche: novel imaging techniques permit in vivo observation and targeted biopsy of limbal epithelial stem cell.. Stem Cells.

[10] Du Y, Funderburgh ML, Mann MM, SundarRaj N, Funderburgh JL (2005). Multipotent Stem Cells in human corneal stroma.. Stem Cells.

[11] de Paiva CS, Chen Z, Corrales RM, Pflugfelder SC, Li DQ (2005). ABCG2 transporter identifies a populationof clonogenic human limbal epithelial cells.. Stem Cells.

[12] Joseph A, Powell-Richards AOR, Shanmuganathan VA, Dua HS (2004). Epithelial cell characteristics of cultured human limbal explants.. Br J Ophthalmol.

[13] Lavker RM, Tseng SC, Sun TT (2004). Corneal epithelial stem cells at the limbus: looking at some old problems from a new angle.. Exp Eye Res.

[14] Dua HS, Joseph A, Shanmuganathan VA, Jones RE (2003). Stem cell differentiation and the effects of deficiency.. Eye (Lond).

[15] Espana EM, Di Pascuale MA, He H, Kawakita T, Raju VK, Liu CY, Tseng SC (2004). Characterization of corneal pannus removed from patients with total limbal stem cell deficiency.. Invest Ophthalmol Vis Sci.

[16] Matic M, Petrov IN, Chen S, Wang C, Dimitrijevich SD, Wolosin JM (1997). Stem cells of the corneal epithelium lack connexins and metabolite transfer capacity.. Differentiation.

[17] Cancelas JA, Koevoet WL, de Koning AE, Mayen AE, Rombouts EJ, Ploemacher RE (2000). Connexin-43 gap junctions are involved in multiconnexin-expressing stromal support of hemopoietic progenitors and stem cells.. Blood.

[18] Rosendaal M, Green CR, Rahman A, Morgan D (1994). Up-regulation of the connexin43+ gap junction network in haemopoietic tissue before the growth of stem cells.. J Cell Sci.

[19] Wen H, Blume PA, Sumpio BE (2009). Role of integrins and focal adhesion kinase in the orientation of dermal fibroblast exposed to cyclic stain.. Int Wound J.

[20] Schreiber TD, Steinl C, Essi M, Abele H, Geiger K, Muller CA, Aicher WK, Klein G (2009). The integrin α9β1 on hematopoietic stem cell progenitor cells: involvement in cell adhesion, proliferation and differentiation.. Haematologica.

[21] Zhou J, Chen F, Xiao J, Li C, Liu Y, Ding Y, Wan P, Wang X, Huang J, Wang Z (2011). Enhanced functional properties of corneal epithelial cells by coculture with embryonic stem cells via the integrin β1-FAK-PI3K/Akt pathway.. Int J Biochem Cell Biol.

[22] Yeung AM, Tint NL, Kulkarni BB, Mohammed I, Suleman H, Hopkinson A, Dua HS (2008). Infant limbus: an immunological study.. Exp Eye Res.

[23] Ljubimov AV, Burgeson RE, Butkowski RJ, Michael AF, Sun TT, Kenney MC (1995). Human corneal basement membrane heterogeneity: topographical differences in the expression of type IV collagen and laminin isoforms.. Lab Invest.

[24] Chen W, Garcia JG, Jacobson JR (2010). Integrin beta4 attenuates SHP-2 and MAPK signaling and reduces human lung endothelial inflammatory responses.. J Cell Biochem.

[25] Dua HS, Miri A, Said DG (2010). Contemporary limbal stem cell transplantation: a review.. Clin Experiment Ophthalmol.

[26] Figueira EC, Di Girolamo N, Coroneo MT, Wakefield D (2007). The phenotype of limbal epithelial stem cells.. Invest Ophthalmol Vis Sci.

[27] Dua HS, Miri A, Alomar T, Yeung AM, Said DG (2009). The role of limbal stem cells in corneal epithelial maintenance: testing the dogma.. Ophthalmology.

